# Topical Application of Adelmidrol + *Trans*-Traumatic Acid Enhances Skin Wound Healing in a Streptozotocin-Induced Diabetic Mouse Model

**DOI:** 10.3389/fphar.2018.00871

**Published:** 2018-08-23

**Authors:** Rosalba Siracusa, Daniela Impellizzeri, Marika Cordaro, Enrico Gugliandolo, Alessio F. Peritore, Rosanna Di Paola, Salvatore Cuzzocrea

**Affiliations:** ^1^Department of Chemical, Biological, Pharmaceutical and Environmental Sciences, University of Messina, Messina, Italy; ^2^Department of Pharmacological and Physiological Sciences, Saint Louis University School of Medicine, St. Louis, MO, United States

**Keywords:** diabetes mellitus, adelmidrol, *trans*-traumatic acid, topical treatment, healing of cutaneous wounds

## Abstract

Impaired wound healing is considered to be one of the severe complications associated with diabetes. Adelmidrol and trans-traumatic acid are commonly called Nevamast^®^. This gel consists precisely of 2% adelmidrol and 1% trans-traumatic acid. Thanks to its components, it is capable of favoring the natural process of skin re-epithelialization. This study tests the theory that topical usage of adelmidrol + trans-traumatic acid has important effects on the healing and closure of diabetic wounds in a streptozotocin (STZ)-induced diabetic mouse model. Diabetes was induced by intraperitoneal injection of STZ (60 mg/kg) in 0.01 M citrate buffer (pH 4.5) administrated for 5 consecutive days. After diabetes induction, two longitudinal incisions were made on the dorsum of the mice. The animals were killed between 6 and 12 days from wound induction. We found that diabetic mice compared to control mice presented: a retarded wound closure, characterized by an important reduction in the levels of transforming growth factor-β, plus an important increase of vascular endothelial growth factor and endothelial-type nitric oxide synthase expression, together with a reduction of adhesion molecules such as intercellular adhesion molecule-1 and P-selectin and a prolonged elevation of the levels of matrix metalloproteinase-9 and matrix metalloproteinase-2 in wound tissues. This study demonstrates that topical application of adelmidrol + trans-traumatic acid has important effects on the healing and closure of diabetic wounds in an STZ-induced diabetic mouse model.

## Introduction

The term “diabetes mellitus” (DM) refers to a group of metabolic dysfunctions characterized by a high concentration of glucose in the blood. Generally, DM is caused by an elaborate interaction between environmental and genetic factors, which leads to inadequate secretion and/or diminished insulin action (Mustoe, 2004; American Diabetes, 2009). DM is among the most diffused endocrine diseases and its percentage of morbidity is constantly increasing. As a consequence of the hyperglycemia that characterizes this pathology, the complications generated can be divided into acute or long term (Brownlee, 2005). The most severe acute complications are hypoglycemic coma, and hyperosmolar and ketoacidotic coma. Long-term complications usually depend on the gravity and duration of diabetes. These changes affect various organs and systems and are responsible for the morbidity and mortality associated with this disease. Furthermore, these complications are arbitrarily divided into vascular and non-vascular (Lachin et al., 2008). Vascular complications include neuropathy, nephropathy, retinopathy, coronary heart sickness, cerebrovascular disorder, and peripheral arterial disease (Cameron et al., 2001). Non-vascular complications, on the other hand, involve different systems including the nervous system, the gastrointestinal tract, the genitourinary system, skin’s ulcerations and scarring problems, and at last retinopathy. In diabetic patients, the incidence of infectious diseases is also increased (Rask-Madsen and Kahn, 2012; Chawla et al., 2016).

In developed countries, one of the most preeminent cases of hospital admissions for patients with diabetes is chronic diabetic foot ulcer, which often induces pain that results in a lower quality of life (Boulton, 2008). It was declared that 15% of the total patients with diabetes develop foot ulceration, 84% of whom undergo amputation (Bartus and Margolis, 2004; Boulton et al., 2005).

Healing of cutaneous wounds is a process that develops through three typical stages: (1) an inflammatory phase that consists of platelet aggregation and recruitment of inflammatory cells to the wound point; (2) a proliferative stage that includes the migration and proliferation of fibroblasts, keratinocytes, and endothelial cells, leading to re-epithelialization and granulation tissue formation; (3) a prolonged remodeling phase (Hubner et al., 1996; Martin, 1997; Singer and Clark, 1999). Despite numerous advances in wound care and management (Sen et al., 2009), skin’s ulcer healing in a diabetic patient is retarded because all stages of the succession of biochemical and cellular events of wound healing (WH) are altered (Brown et al., 1992; Shukla et al., 1998; Mustoe, 2004). In particular, WH in DM is delayed because of compromised angiogenesis, inadequate blood flow, increased inflammation, reduced proliferation of fibroblasts (Hehenberger et al., 1999), and reduced re-epithelialization by keratinocytes (Stadelmann et al., 1998; Mansbridge et al., 1999; Sheetz and King, 2002). Despite the fact that some therapeutic methods, such as treatment with recombinant growth factors and gene therapy, have been used targeting to stimulate angiogenesis, these methods have restrictions, such as care issues and high costs (Ko et al., 2011). A pharmaceutical method could be the most effective and strategic method, particularly in terms of cost, convenience, and safety. Nevamast^®^ has been formulated as isosmotic hydrophilic gel, and it is compatible with biologic exudates and consists of 2% adelmidrol and 1% trans-traumatic acid. Due to the effect of its components, it favors the natural re-epithelialization process of skin lesions even deeper. The presence of *trans*-traumatic acid causes optimization of the natural convergence process of keratinocytes leading to a progressive normalization of trans-epidural water loss and a barrier effect capable of containing the risk of being colonized by microorganisms. Adelmidrol, a lipid substance widely used as an adjuvant in normalization of dermoepidermal inflammatory processes, promotes the physiological endogenous increase of local levels of palmitoylethanolamide (PEA), necessary for the control of the local neurovasal equilibrium and the consequent optimal tissue repair (Pulvirenti et al., 2007). Therefore, the association of the two main substances determines, in consideration of the respective mechanism of clearly differentiated action, a synergistic effect able to favor the natural process of skin re-epithelialization (Della Valle et al., 2016). In the following study, therefore, we have evaluated the effects of treatment with adelmidrol + *trans*-traumatic acid on WH processes in diabetic mice and whether this treatment was capable or not of accelerating wound’s healing.

## Materials and Methods

### Animals

Male adult CD1 mice (25–30 g; Envigo, Italy) were accommodated in a controlled habitat and provided with water and ordinary rodent food. Mice were located in stainless steel cages in a room kept at 22 ± 1°C with a 12-h dark, 12-h light cycle. The animals were familiarized to their habitat for 1 week and had access to rodent standard diet and water *ad libitum*. Animal care was in accordance with the novel legislation for the protection of animals used for scientific purposes (D.Lgs 2014/26 and EU Directive 2010/63).

### Diabetes Induction and Experimental Wound Healing

Diabetes was induced by intraperitoneal injection of STZ (60 mg/kg) in 0.01 M citrate buffer (pH 4.5) daily for 5 consecutive days, during the fasting state. Control animals received equivalent doses of the citrate buffer solution. Hyperglycemia happened 2 days after STZ injection and was checked using an Accu-Check Active glucometer (Roche, Lyon, France). Animals were considered to have diabetes when blood glucose was ≥300 mg/dL (i.e., 16.6 mmol/L) 48 h after the STZ injection. The animals were housed for 2 weeks before wound formation and 2% adelmidrol and 1% trans-traumatic acid (Nevamast^®^) treatment. After diabetes induction, mice were anesthetized with intraperitoneal xylazine and ketamine (0.16 and 2.6 mg/kg body weight, respectively), hair on the back was clean-shaven by a depilatory cream, and the skin was lapped with a povidone–iodine solution and then cleaned with sterile water. Two full-thickness longitudinal cuts (4 cm) were made on the dorsum of the animals, and the wound boundaries were closed with surgical sutures (4-0 silk) at 1-cm intervals. Ten mice for each group were killed after 6 and 12 days, respectively, and the wounds were separated into two segments. The first one was used for histology and the second one for molecular analysis.

#### Experimental Groups

The animals were randomly distributed into the following groups:

##### Group 1: Control + Vehicle 6 days (*N* = 10)

Animals received intraperitoneal injection of STZ (60 mg/kg in 0.01 M citrate buffer pH 4.5) for 5 days. After 15 days from the last intraperitoneal injection of STZ, mice were not subjected to the wound surgical procedure and topically treated with placebo solution daily for 6 days.

##### Group 2: Control + Vehicle 12 days (*N* = 10)

Animals received intraperitoneal injection of STZ (60 mg/kg in 0.01 M citrate buffer pH 4.5) for 5 days. After 15 days from the last intraperitoneal injection of STZ, mice were not subjected to the wound surgical procedure and topically treated with placebo solution daily for 12 days.

##### Group 3: Control + Adelmidrol + *Trans*-traumatic Acid 6 days (*N* = 10)

Animals received intraperitoneally administered injection of STZ (60 mg/kg in 0.01 M citrate buffer pH 4.5) for 5 days. After 15 days from the last intraperitoneal injection of STZ, mice were not subjected to the wound surgical procedure and topically treated with adelmidrol and trans-traumatic acid daily for 6 days (data not shown).

##### Group 4: Control + Adelmidrol + *Trans*-traumatic acid 12 days (*N* = 10)

Animals received intraperitoneally administered injection of STZ (60 mg/kg in 0.01 M citrate buffer pH 4.5) for 5 days. After 15 days from the last intraperitoneal injection of STZ, mice were not subjected to the wound surgical procedure and topically treated with adelmidrol and trans-traumatic acid daily for 12 days (data not shown).

##### Group 5: WH + Vehicle 6 days (*N* = 10)

Mice received intraperitoneal injections of STZ (60 mg/kg in 0.01 M citrate buffer pH 4.5) for 5 days. After 15 days from the last intraperitoneal injection of STZ, mice were subjected to WH and topically treated with placebo solution daily for 6 days.

##### Group 6: WH + Vehicle 12 days (*N* = 10)

Mice received intraperitoneal injections of STZ (60 mg/kg in 0.01 M citrate buffer pH 4.5) for 5 days. After 15 days from the last intraperitoneal injection of STZ, mice were subjected to WH and topically treated with placebo solution daily for 12 days.

##### Group 7: WH + Adelmidrol + *Trans*-traumatic acid 6 days (*N* = 10)

Mice received intraperitoneal injections of STZ (60 mg/kg in 0.01 M citrate buffer pH 4.5) for 5 days. After 15 days from the last intraperitoneal injection of STZ, mice were subjected to wound procedure and topically treated with adelmidrol and trans-traumatic acid daily for 6 days.

##### Group 8: WH + Adelmidrol + *Trans*-traumatic acid 12 days (*N* = 10)

Mice received intraperitoneal injections of STZ (60 mg/kg in 0.01 M citrate buffer pH 4.5) for 5 days. After 15 days from the last intraperitoneal injection of STZ, mice were subjected to wound procedure and topically treated with adelmidrol and trans-traumatic acid daily for 12 days.

At 6 and 12 days after WH, mice were killed by anesthetic (xylazine and ketamine) overdose.

### Analytic Methods

Blood glucose and weight of all mice were tested before killing. Blood samples were collected before diabetic induction, 2 weeks after diabetes induction, and 0, 6, and 12 days after wounding. Animals were fasted for at least 16 h before blood glucose measurement procedures. Water remained freely available throughout the entire fasting period. Food was returned after the collection of the final blood sample. A drop of blood was taken from mice by nicking the tail tip with a blade. Hyperglycemia was checked using an Accu-Check Active glucometer (Roche).

### Light Microscopy

For histologic examination, ordinary hematoxylin and eosin (H&E) stain was performed. Briefly, 6 and 12 days after the incision, animals in every group were selected and killed after anesthesia. Wound tissue specimens were quickly removed and were fixed in 4% formalin for at least 24 h at room temperature. After dehydration with ethanol and fixing with paraffin, 7 μm sections were prepared and subsequently stained with H&E. Wound tissue specimens were then observed under a light microscope (Leica QWin V3, Cambridge, United Kingdom). Sections were scored according to Asadi et al. (2013). All the histological analyses were executed in a blinded manner. Galeano et al. (2004) described previously the criteria that are used as a histological score of WH. Sections were also stained by Masson’s trichrome method for collagen detection, and observed under an optical microscope. The degree of fibrosis was estimated as percentage of fibrotic area (blue staining) and quantified by the Image J 1.8.0 software.

### Staining of Mast Cells

Identification of mast cells (MCs) was executed in wound tissue specimens’ sections by blue toluidine stain as described previously (Ahmad et al., 2012).

### Immunohistochemical Localization of TGF-β, VEGF, ICAM-1, and P-selectin

Immunohistochemical evaluation for transforming growth factor (TGF)-β, vascular endothelial growth factor (VEGF), intercellular adhesion molecule 1 (ICAM-1), and P-selectin was realized as previously described (Siracusa et al., 2017). Slices were incubated overnight with an anti-TGF-β mouse monoclonal antibody (Santa Cruz Biotechnology; 1:100 in phosphate-buffered saline [PBS], v/v), anti-VEGF mouse monoclonal antibody (Santa Cruz Biotechnology; 1:100 in PBS, v/v), anti-ICAM-1 mouse monoclonal antibody (Santa Cruz Biotechnology; 1:100 in PBS, v/v), and anti-P-selectin mouse monoclonal antibody (Santa Cruz Biotechnology; 1:100 in PBS, v/v). Sections were washed with PBS and incubated with a secondary antibody. Specific category was recognized with a biotin-conjugated goat anti-rabbit IgG and avidin–biotin peroxidase complex (Vector Labs Inc., Burlingame, CA, United States). To verify the binding specificity for different antibodies, some sections were also incubated with only a primary or secondary antibody; no positive staining was observed in these sections. The slices were quantitatively assessed for a modification in immunoreactivity by computer-assisted color image examination (Leica QWin V3, Cambridge, United Kingdom). The proportion area of immunoreactivity (definite by the number of positive pixels) was expressed as percent of entire tissue area (red staining). Histochemical stain were obtained from each animal in each experimental group. All the immunocytochemistry analysis was carried out without knowledge of the treatments.

### Western Blot Analysis

To execute western blot analysis, the animals were killed by anesthetic overdose (ketamine 2.6 and xylazine 0.16 mg/kg body weight). Wound tissue specimens of each mouse were quickly removed; placed in extraction Buffer A comprising 20 mM leupeptin, 0.15 mM pepstatin A, 1 mM sodium orthovanadate, and 0.2 mM phenylmethylsulfonyl fluoride (PMSF); homogenized for 2 min; and centrifuged at 4°C at 12,000 rpm for 4 min. Supernatants represented the cytosolic portion. The pellets, which contain nuclei, were resuspended in Buffer B comprising 10 mM Tris–HCl pH 7.4, 150 mM NaCl, 1 mM EGTA, 1% Triton X-100, 1 mM EDTA, 0.2 mM PMSF, 20 mM leupeptin, and 0.2 mM sodium orthovanadate, and centrifuged for 10 min at 4°C at 12,000 rpm. Protein concentrations were measured by the Bio-Rad protein assay. Bovine serum albumin has been used as reference. The expression of matrix metalloproteinase (MMP)-9, MMP-2, endothelial nitric oxide synthase (eNOS), and VEGF were quantified in cytosolic fractions. The filters were probed with specific antibodies for an anti-MMP-9 mouse monoclonal antibody (1:500; Santa Cruz Biotechnology), anti-MMP-2 rabbit polyclonal antibody (1:1000; Abcam), anti-eNOS mouse monoclonal antibody (1:1000; Santa Cruz Biotechnology), and anti-VEGF mouse monoclonal antibody (1:1000; Santa Cruz Biotechnology); were mixed in 1X PBS, 5% w/v non-fat desiccated milk, 0.1% Tween-20; and incubated overnight at 4°C. Membranes were then incubated with peroxidase-conjugated goat anti-rabbit IgG or peroxidase-conjugated bovine anti-mouse IgG secondary antibody (1:2000, Jackson ImmunoResearch) for 1 h out of the fridge. To assess that blots were loaded with uniform volumes of protein lysates, they were also incubated with a β-actin antibody (1:5000; Santa Cruz Biotechnology) for cytosolic proteins or a lamin A/C antibody (1:5000; Santa Cruz Biotechnology) for nuclear proteins. Signals were detected with a Super Signal West Pico Chemiluminescent Substrate according to the manufacturer’s instructions (Pierce Thermo Scientific, Rockford, IL, United States). The relative expression of protein bands was quantified by densitometric with BIORAD ChemiDocTMXRS+ software and standardized to β-actin levels. Images of blot signals (8 bits/600 dpi resolution) were transmitted to analysis software (Image Quant TL, v2003).

### Materials

Adelmidrol + trans-traumatic acid (Nevamast^®^ gel) was obtained from Epitech group S.p.A (Saccolongo, Italy). All other chemicals were obtained from commercial sources and were of the highest grade accessible. All stock solutions were made in non-pyrogenic saline (0.9% NaCl, Baxter, Milan, Italy).

### Statistical Evaluation

All values in the illustrations and the text are expressed as mean ± SEM. Data presented in the figures are representative of at least three experiments performed on diverse *in vivo* experimental days. In each experiment, we used five animals per group, unless otherwise indicated. Data were examined by one-way or two-way analysis of variance followed by a Bonferroni *post hoc* test for multiple comparisons. A *p*-value of less than 0.05 was considered significant.

## Results

### Blood Analysis and Changes in Animal Weight After Inducing Diabetes

We measured the body weight and blood glucose increase in mice before and throughout the indicated time points after WH. Data collected from 10 animals taken from each group revealed that all mice exhibited an important elevation in the glucose levels and an increase in the body weight in all experimental groups (**Table 1**).

**Table 1:** Blood analysis and animal weight.

	Control + Vehicle	WH + Vehicle	WH + Adelmidrol + *Trans*-traumatic acid
**Blood Glucose Level (mg/dL)**			
Onset of diabetic induction	113 ± 9	119 ± 10	114 ± 7^xxx^
Two weeks after diabetes induction	374 ± 11^∗∗∗^	317 ± 11^∘∘∘^	355 ± 15^§§§^
0 day after wounding	433 ± 14^∗∗∗^	421 ± 11^∘∘∘^	440 ± 18^§§§^
6 days after wounding	544 ± 8^∗∗∗^	538 ± 7^∘∘∘^	536 ± 13^§§§^
12 days after wounding	546 ± 10^∗∗∗^	532 ± 17^∘∘∘^	562 ± 16^§§§^
**Body weight increase (gr)**			
Onset of diabetic induction	26,56 ± 1,40	26,16 ± 2,07	25,94± 2,02
Two weeks after diabetes induction	32,20 ± 1,64^∗∗∗^	34,44 ± 3,19^∘∘∘^	36,76 ± 0,82^§§§^
0 day after wounding	35,28 ± 0,79^∗∗∗^	38,58 ± 1,12^∘∘∘^	38,64 ± 0,60^§§§^
6 days after wounding	39,76 ± 0,46^∗∗∗^	40,62 ± 0,83^∘∘∘^	40,76 ± 0,77^§§§^
12 days after wounding	42,38 ± 0,62^∗∗∗^	42,04 ± 0,94^∘∘∘^	41,74 ± 0,40^§§§^


*Blood glucose and body weight in the three groups of mice from the onset of diabetic induction until the finish of the experiments. Blood glucose level (mg/dL): ^∗∗∗^*p* < 0.001 vs. Control + Vehicle at the time of diabetic induction; ^∘∘∘^*p* < 0.001 vs. WH + Vehicle at the time of diabetic induction; ^§§§^*p* < 0.001 vs. WH + Adelmidrol + *Trans*-traumatic acid at the time of diabetic induction. Body weight increase (gr): ^∘∘∘^*p* < 0.001 vs. WH + Vehicle at the time of diabetic induction; ^§§§^*p* < 0.001 vs. WH+ Adelmidrol + Trans-traumatic acid at the time of diabetic induction.*

### Effect of Adelmidrol + *Trans*-Traumatic Acid on Tissue Repair

**FIGURE 1:**
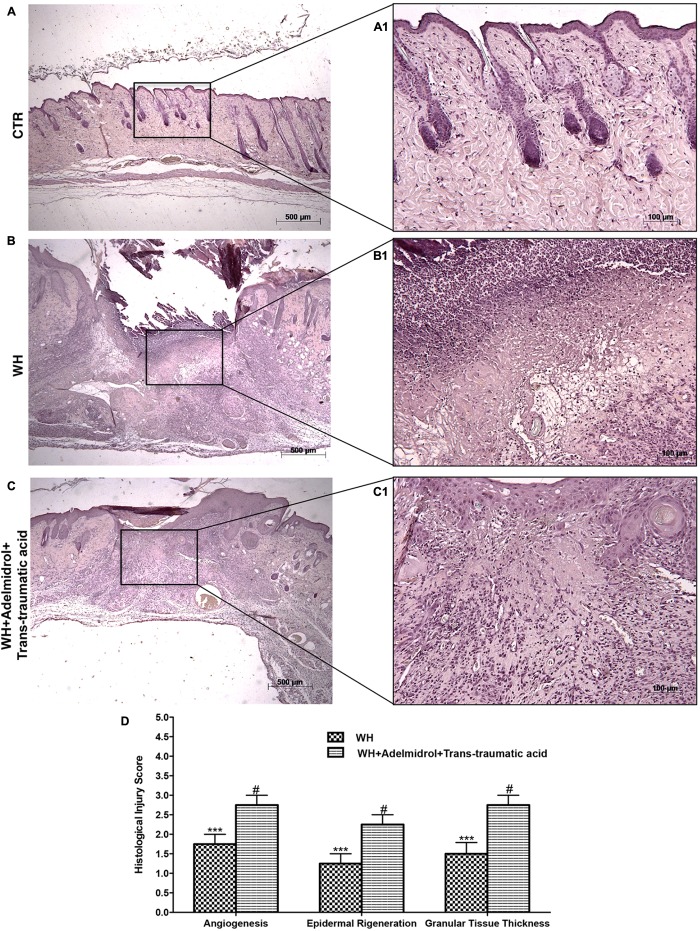
Effects of adelmidrol + *trans*-traumatic acid on histological parameters after 6 days from wound induction. Diabetic control mice showed a normal structure of skin and no injury **(A,A1)**, whereas 6 days after wounding, diabetic mice showed significant alteration of angiogenesis, epidermal regeneration, and granular tissue thickness **(B,B1)**. Adelmidrol + *trans*-traumatic acid treatment has almost completely restored skin structure **(C,C1)**. Figures are representative of at least three separate experiments. The histological score summarizes these data **(D)**. ^∗∗∗^*p* < 0.001 vs. Control; #*p* < 0.05 vs. WH + Vehicle, as determined using one-way ANOVA followed by the Bonferroni *post hoc* test.

**FIGURE 2:**
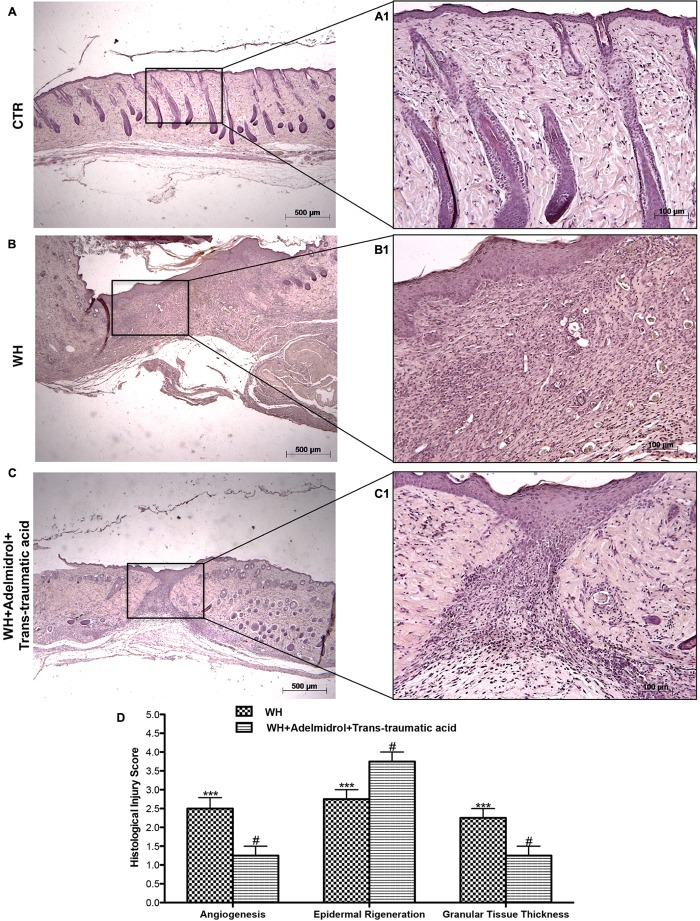
Effects of adelmidrol + *trans*-traumatic acid on histological parameters after 12 days from wound induction. Diabetic mice of the control group showed a normal structure of skin and no injury **(A,A1)**, whereas 12 days after wounding, diabetic mice showed significant alteration of angiogenesis, epidermal regeneration, and granular tissue thickness **(B,B1)**. Adelmidrol + *trans*-traumatic acid treatment restored skin structure favoring the re-epithelization **(C,C1)**. Figures are representative of at least three separate experiments. The histological score summarizes these data **(D)**. ^∗∗∗^*p* < 0.001 vs. Control; #*p* < 0.05 vs. WH + Vehicle, as determined through one-way ANOVA followed by the Bonferroni *post hoc* test.

Galeano et al. (2001) previously demonstrated that in skin-wound models, the impairment of WH is characterized by a reduction in angiogenesis, retarded formation of granulation tissue, diminished collagen content, reduced arteriolar quantity and density, loss of vasculature tone, and reduction in the cross-sectional area of novel vessel walls. **Figure 1** shows tissue injury of wounds after 6 days of wound induction, whereas **Figure 2** shows the trend of tissue damage 12 days after wound’s induction. Mice of the WH+Vehicle group (**Figures 1B,B1** and relative histological analysis in **Figure 1D**; and **Figures 2B,B1** and relative histological analysis in **Figure 2D**) had a significant impaired wound characterized by a great number of neutrophils that were mixed with fibroblasts in an edematous tissue, and limited dermal and epidermal organization compared with the Control + Vehicle group (**Figures 1A,A1** and relative histological analysis in **Figure 1D**; and **Figures 2A,A1** and relative histological analysis in **Figure 2D**). In diabetic mice exposed to wound and treated with adelmidrol + *trans*-traumatic acid, re-epithelialization was accelerated after 6 days (**Figures 1C,C1** and relative histological analysis in **Figure 1D**) to be complete after 12 days (**Figure 2C,C1** and relative histological analysis in **Figure 2D**), showing normal differentiation and keratinization with epidermal elongation extending over two-thirds of the wound surface and modest glycogen storage to the margins of the wound area. The granulation tissue was apparently well organized and dermal redevelopment was characterized by granulation tissue rich in fibroblasts. In addition, the degree of infiltrated inflammatory cells after adelmidrol + *trans*-traumatic acid treatment was minimal, with few polymorphonuclear cells scattered in the wound area and around the vessels compared with the WH + Vehicle group (**Figures 1C,C1** and relative histological analysis in **Figure 1D**; and **Figures 2C,C1** and relative histological analysis in **Figure 2D**).

### Adelmidrol + *Trans*-Traumatic Acid Effects on Collagen Fibers in the Wounded Skin

**FIGURE 3:**
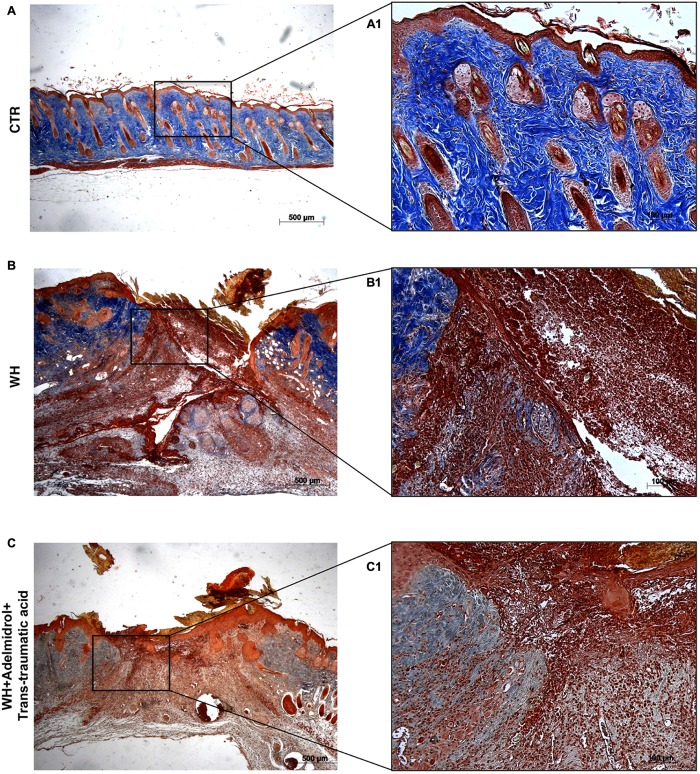
Effect of adelmidrol + *trans*-traumatic acid treatment on Masson’s trichrome after 6 days from damage. Masson trichrome staining showed that skin tissues from the control group displayed collagen fibers **(A,A1)**, which reduced in the WH + Vehicle group **(B,B1)**. After 6 days of the injury, adelmidrol + *trans*-traumatic acid treatment showed a stimulating action on collagen arrangement **(C,C1)**. Figures are representative of at least three separate experiments.

**FIGURE 4:**
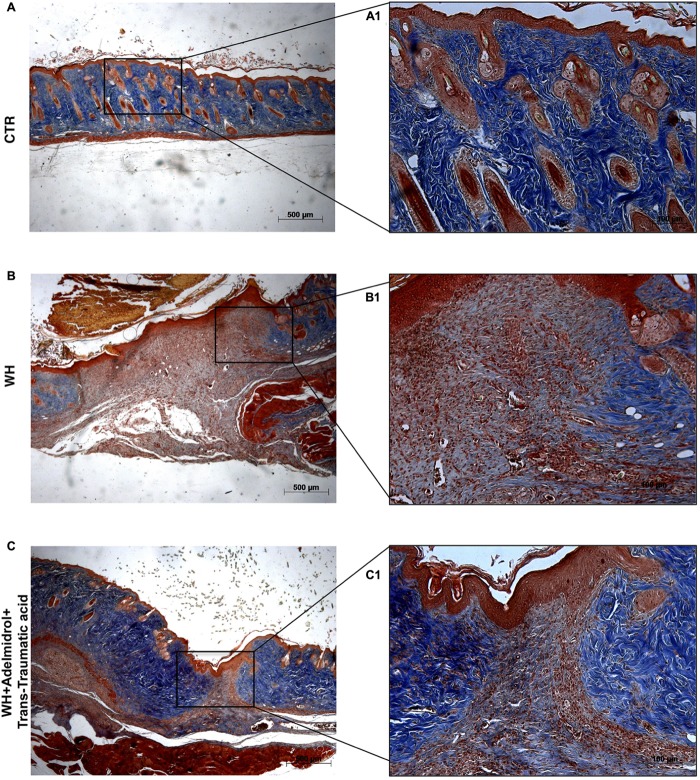
Effect of adelmidrol + *trans*-traumatic acid treatment on Masson’s trichrome after 12 days from damage. Masson trichrome staining showed that skin tissues from the control group displayed collagen fibers **(A,A1)**, which reduced in the WH + Vehicle group **(B,B1)**. After 12 days of the injury, adelmidrol + *trans*-traumatic acid treatment showed a stimulating action on collagen arrangement **(C,C1)**. Figures are representative of at least three separate experiments.

To confirm substrate formation in the dermis, Masson’s trichrome staining was performed. This staining showed clear visible fine and coarse collagen deposition and its arrangement in the wounded skin. About that, control groups showed collagen fibers (**Figures 3A,A1**, **4A,A1**), whereas WH + Vehicle groups showed a significant reduction of collagen fibers both on the 6th and 12th day after wound induction (**Figures 3B,B1**, **4B,B1**). Furthermore, Masson’s trichrome stain showed a stimulating action of adelmidrol + *trans*-traumatic acid on collagen arrangement. Collagen synthesis was observed already on the sixth day; in fact, a clear blue stain was identified around the wound (**Figures 3C,C1**). Instead, in the mice treated for 12 days with adelmidrol + *trans*-traumatic acid, a more intense stain was observed indicating a greater synthesis of collagen around the wound (**Figures 4C,C1**). Figures are representative of at least three separate experiments.

### Adelmidrol + *Trans*-Traumatic Acid Effects on MC Infiltration After Wound Induction

**FIGURE 5:**
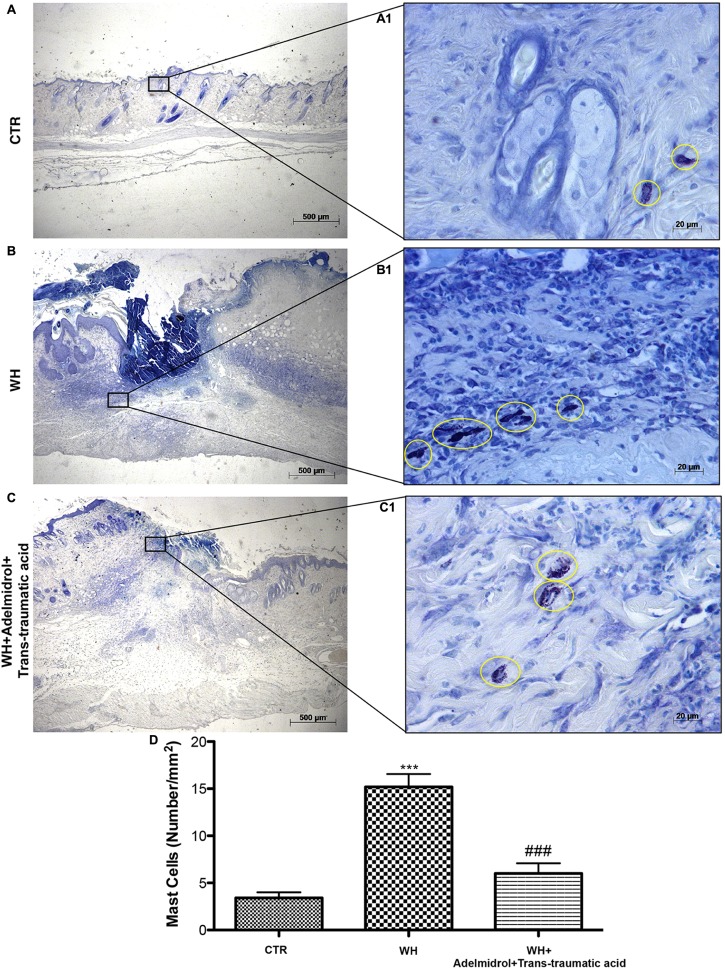
Effects of adelmidrol+trans-traumatic acid on mast cell degranulation in skin after 6 days from wound induction. Toluidine blue staining was used to identify mast cell infiltration (encircled), characterized by dark lilac blue granules: **(A,A1)** Control group; **(B,B1)** WH + Vehicle group; **(C,C1)** WH + Adelmidrol + *Trans*-traumatic acid group. **(D)** Mast cell number per unit area of tissue (mast cell density). Figures are representative of at least three separate experiments. Values are means ± SEM of 10 animals for each group. ^∗∗∗^*p* < 0.001 vs. Control, ###*p* < 0.001 vs. WH + Vehicle.

**FIGURE 6:**
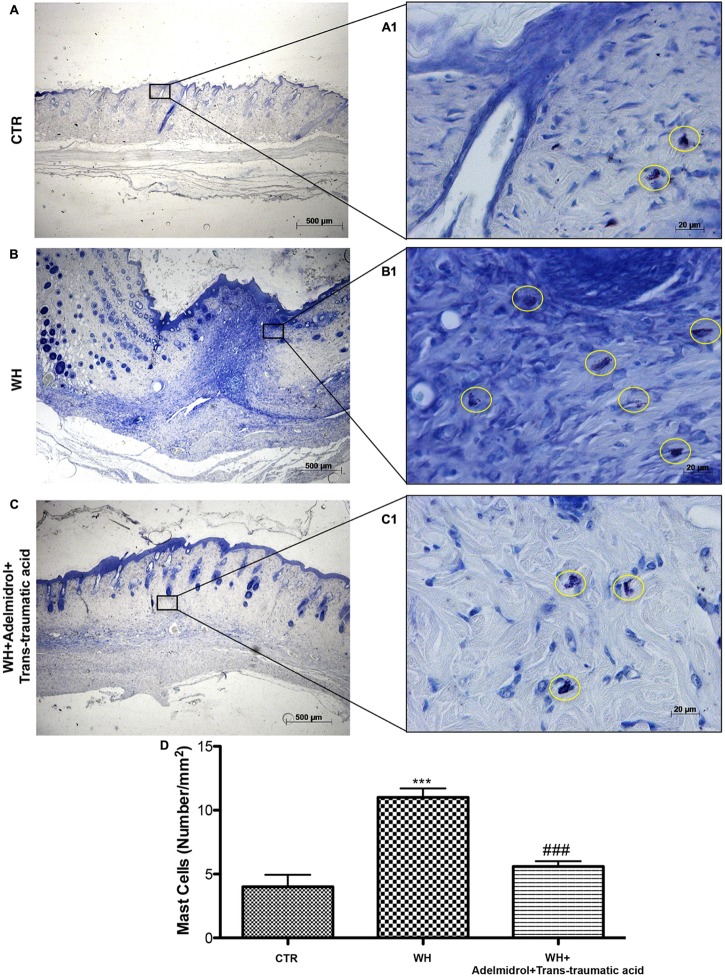
Effects of adelmidrol + *trans*-traumatic acid on mast cell degranulation in skin after 12 days from wound induction. Toluidine blue staining was used to identify mast cell infiltration (encircled), characterized by dark lilac blue granules: **(A,A1)** Control group; **(B,B1)** WH + Vehicle group; **(C,C1)** WH + Adelmidrol + *Trans*-traumatic acid group. **(D)** Mast cell number per unit area of tissue (mast cell density). Figures are representative of at least three separate experiments. Values are means ± SEM of 10 animals for each group. ^∗∗∗^*p* < 0.001 vs. Control, ###*p* < 0.001 vs. WH + Vehicle.

The presence of MCs was used to evaluate the degree of wound inflammation. For this analysis, wound tissue samples were stained with toluidine blue. An important increase in the number of MCs was observed both 6 and 12 days after wound procedure (**Figures 5B,B1** and relative histological analysis in **Figure 5D**; **Figure 6B,B1** and relative histological analysis in **Figure 6D**, respectively) compared with control groups (**Figures 5A,A1** and relative histological analysis in **Figure 5D**; **Figures 6A,A1** and relative histological analysis in **Figure 6D**, respectively). The topical treatment of wounds with adelmidrol + *trans*-traumatic acid significantly reduced the MC infiltration at 6 and 12 days (**Figures 5C,C1** and relative histological analysis in **Figure 5D**; **Figures 6C,C1** and relative histological analysis in **Figure 6D**, respectively).

### Adelmidrol + *Trans*-Traumatic Acid Effects on TGF-β Expression

**FIGURE 7:**
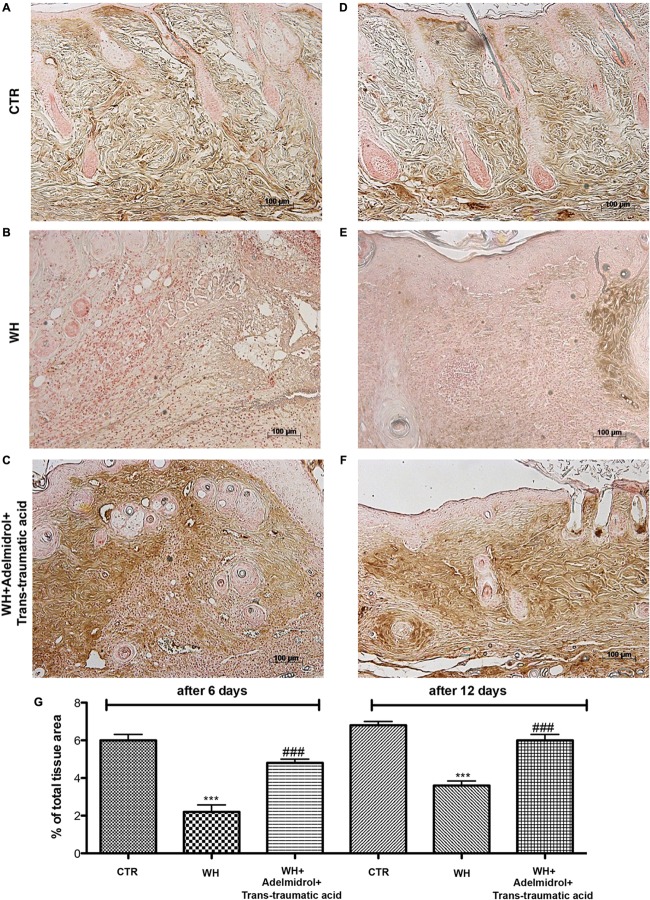
Effects of adelmidrol + *trans*-traumatic acid on levels of TGF-β in skin after 6 and 12 days from wound. Immunohistochemical analysis showed positive staining for TGF-β in the Control group **(A,D)**. Decreased TGF-β expression was observed in skin collected from the WH + Vehicle group 6 and 12 days after injury **(B,E)**, whereas high levels of this protein were found in mice treated with adelmidrol + *trans*-traumatic acid **(C,F)**. The data are also presented graphically as percentage of total tissue area **(G)**. **(G)** ^∗∗∗^*p* < 0.001 vs. Control; ###*p* < 0.001 vs. WH + Vehicle, as determined through one-way ANOVA followed by the Bonferroni *post hoc* test.

TGF-β is a family of growth factors involved in several of essential cellular functions. To investigate the role of TGF-β in the process of both WH and scar formation, immunohistochemical analysis was performed. Either 6 or 12 days after the damage, a high TGF-β expression was found in the Sham group (**Figures 7A,D**; see relative histological analysis in **Figure 7G**). In the WH + Vehicle group, we observed a reduction of TGF-β expression (**Figures 7B,E**; see relative histological analysis in **Figure 7G**), whereas the treatment with adelmidrol + *trans*-traumatic acid significantly increased TGF-β levels (**Figures 7C,F**; see relative histological analysis in **Figure 7G**).

### Effect of Adelmidrol + *Trans*-Traumatic Acid on the Expression of eNOS and VEGF at the 6th and 12th Day After Wound Induction

**FIGURE 8:**
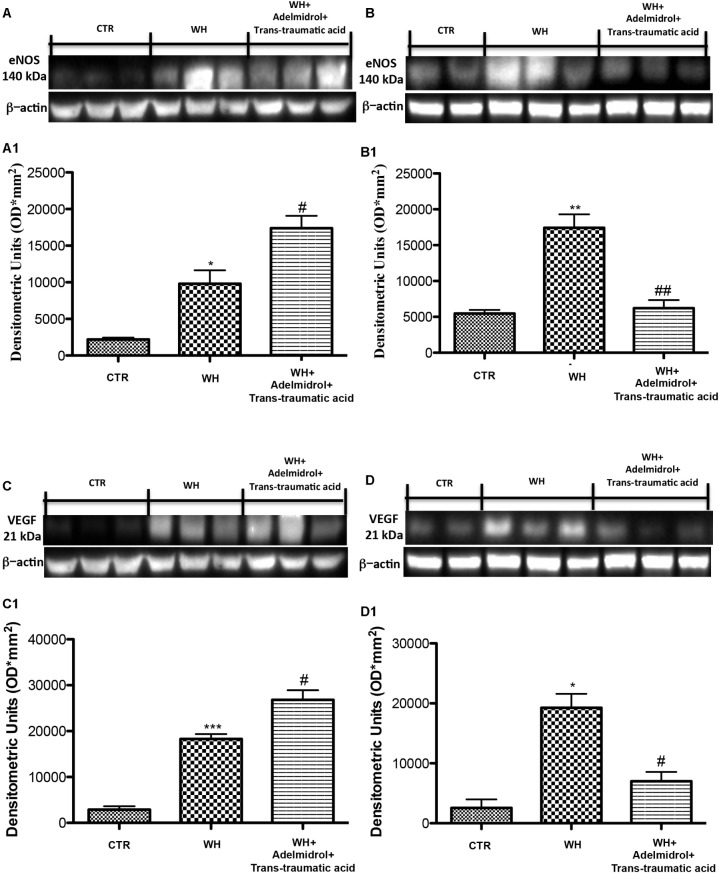
Effects of adelmidrol + *trans*-traumatic acid on eNOS and VEGF expression in diabetic mice after 6 and 12 days from wound induction. Representative western blots showing the effects of adelmidrol + *trans*-traumatic acid on: **(A,A1)** eNOS expression, **(C,C1)** VEGF expression at 6 days after wounding. Adelmidrol + *trans*-traumatic acid treatment increased eNOS and VEGF levels. Data are illustrative of at least three independent experiments. **(A1)** ^∗^*p* < 0.05 vs. Control; #*p* < 0.05 vs. WH + Vehicle; **(C1)** ^∗∗∗^*p* < 0.001 vs. Control; #*p* < 0.05 vs. WH + Vehicle. Western blot analysis showed the effects of adelmidrol + *trans*-traumatic acid on: **(B,B1)** eNOS expression, **(D,D1)** VEGF expression at 12 days after wound induction. Adelmidrol + *trans*-traumatic acid treatment reduced eNOS and VEGF expression. Data are demonstrative of at least three independent experiments. Shown is an illustrative blot of lysates from 10 animals/group, together with a densitometric analysis for all animals. **(B1)** ^∗∗^*p* < 0.01 vs. Control; ##*p* < 0.01 vs. WH + Vehicle; **(D1)** ^∗^*p* < 0.05 vs. Control; #*p* < 0.05 vs. WH + Vehicle. One-way ANOVA followed by the Bonferroni *post hoc* test analyzed data. b-Actin loading control is reused in this figure and in **Figure 12**.

**FIGURE 9:**
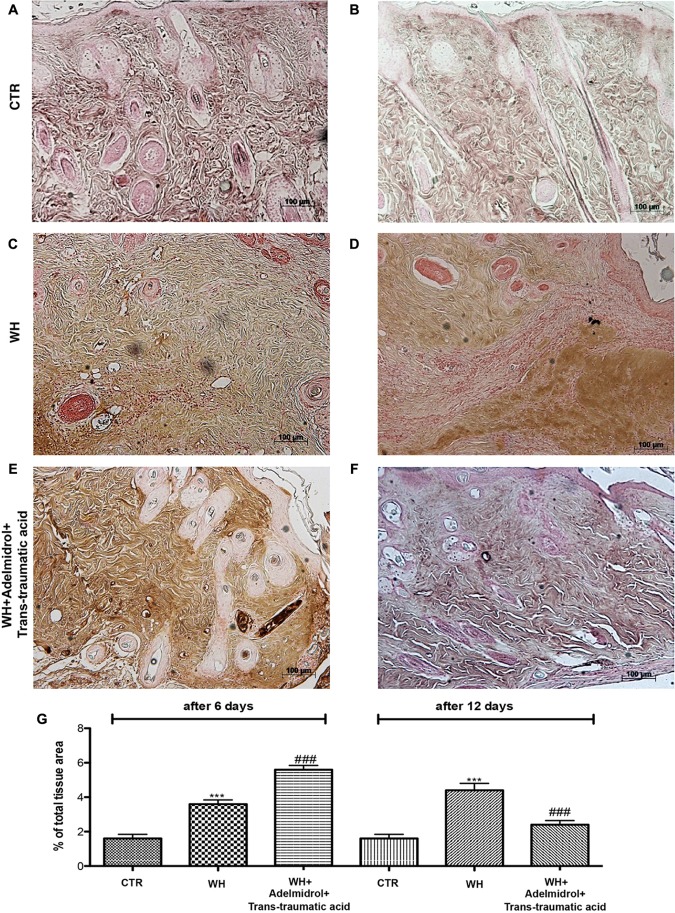
Effects of adelmidrol + trans-traumatic acid on levels of VEGF in diabetic mice after 6 and 12 days from wound induction. At 6 days after wounding, immunohistochemical analysis showed no staining for VEGF in the Control group **(A)**. Increased VEGF expression was observed in skin collected from the WH + Vehicle group 6 days after injury **(B)**. Furthermore, high levels of this protein were found in mice treated with adelmidrol + *trans*-traumatic acid **(C)**. At 12 days after wounding, immunohistochemical analysis showed no staining for VEGF in the Control group **(D)**. Increased VEGF expression was observed in skin collected from the WH + Vehicle group 6 days after injury **(E)**, whereas low levels of VEGF were found in mice treated with adelmidrol + trans-traumatic acid **(F)**. The data are also presented graphically as percentage of total tissue area **(G)**. **(G)** ^∗∗∗^*p* < 0.001 vs. Control; ###*p* < 0.001 vs. WH + Vehicle, as determined through one-way ANOVA followed by the Bonferroni *post hoc* test.

To investigate the mechanisms through which neovascularization is promoted, after the treatment with adelmidrol + *trans*-traumatic acid, we evaluated the levels of eNOS and VEGF. Western blot analysis showed that at 6 and 12 days after wounding, the levels of these proteins were significantly augmented in the vehicle group compared with the Sham group (**Figures 8A,B** for eNOS and see relative densitometric analysis in **Figures 8A1,B1**; **Figures 8C,D** for VEGF and see relative densitometric analysis in **Figures 8C1,D1**). At 6 days after wounding, the treatment with adelmidrol + *trans*-traumatic acid further increased the eNOS and VEGF expression (**Figures 8A,C**; see relative densitometric analysis in **Figures 8A1,C1**), whereas at 12 days after wounding, the levels of these proteins were markedly decreased after treatment with adelmidrol + *trans*-traumatic acid (**Figures 8B,D**; see relative densitometric analysis in **Figures 8B1,D1**). In addition, we investigated the expression of VEGF by immunohistochemical staining. At 6 days after wounding, an increase in VEGF positive stain was found in the vehicle group (**Figure 9B** and see relative histological analysis in **Figure 9G**) compared with the Sham group (**Figure 9A** and see relative histological analysis in **Figure 9G**). Treatment with adelmidrol + *trans*-traumatic acid was able to further increase VEGF positive staining (**Figure 9C** and see relative histological analysis in **Figure 9G**). Instead, at 12 days after wounding, we observed that VEGF levels were still increasing in the vehicle group (**Figure 9E** and see relative histological analysis in **Figure 9G**) compared with control animals (**Figure 9D** and see relative histological analysis in **Figure 9G**), whereas VEGF expression significantly decreased after adelmidrol + *trans*-traumatic acid treatment (**Figure 9F** and see relative histological analysis in **Figure 9G**).

### Effect of Adelmidrol + *Trans*-Traumatic Acid on ICAM-1 and P-selectin Expression After Wound Induction

**FIGURE 10:**
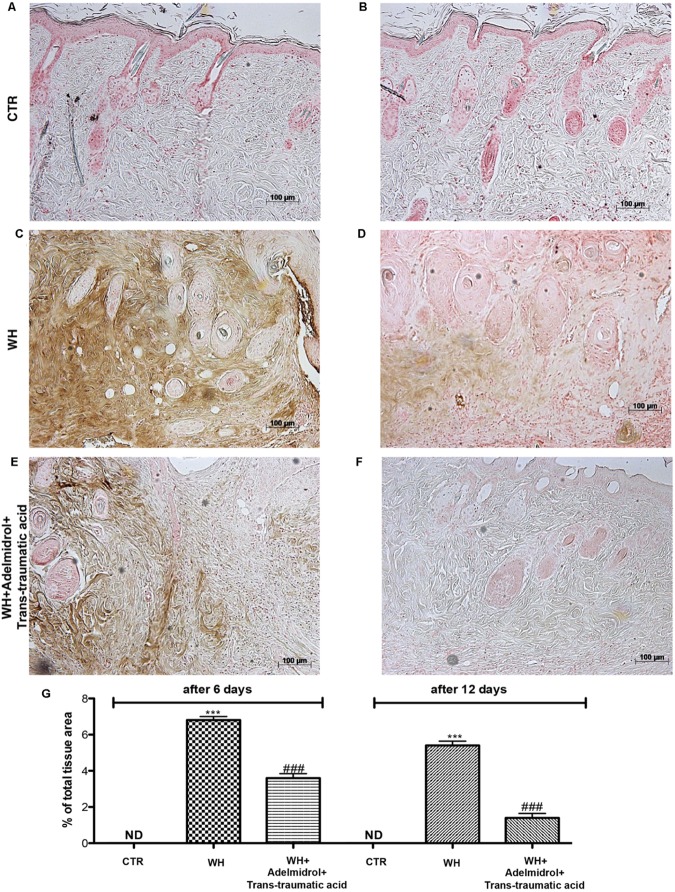
Effects of adelmidrol + trans-traumatic acid on levels of ICAM-1 in skin after 6 and 12 days from wound. Immunohistochemical analysis showed no staining for ICAM-1 in the Control group **(A,D)**. Increased ICAM-1 expression was observed in skin collected from the WH + Vehicle group 6 and 12 days after injury **(B,E)**, whereas low levels of this protein were found in mice treated with adelmidrol + *trans*-traumatic acid **(C,F)**. The data are also presented graphically as percentage of total tissue area **(G)**. **(G)** ^∗∗∗^*p* < 0.001 vs. Control; ###*p* < 0.001 vs. WH + Vehicle; as determined through one-way ANOVA followed by the Bonferroni *post hoc* test.

**FIGURE 11:**
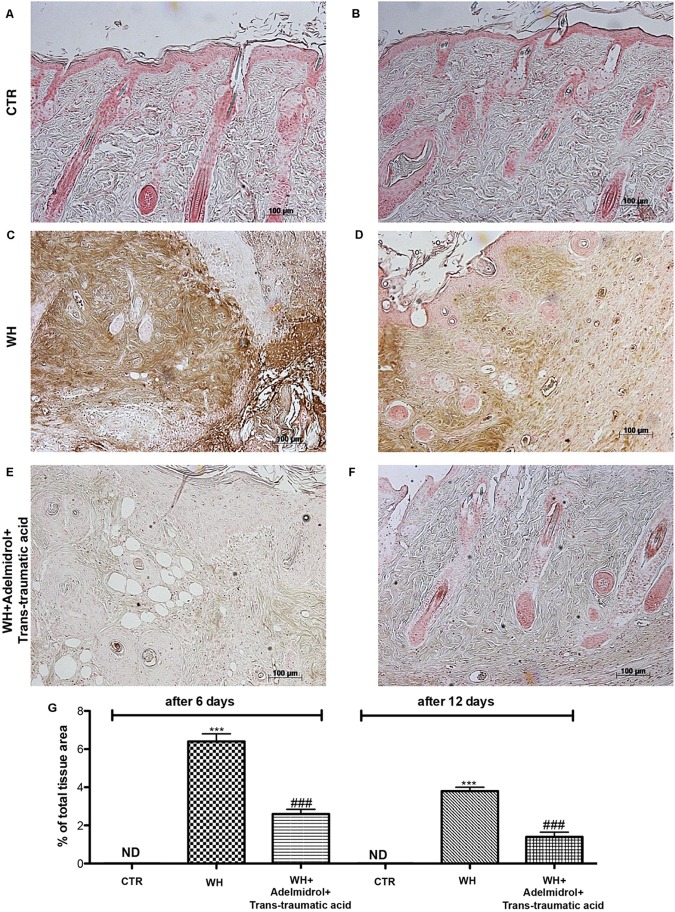
Effects of adelmidrol + *trans*-traumatic acid on levels of P-selectin in skin after 6 and 12 days from wound. Immunohistochemical analysis showed no staining for P-selectin in the Control group **(A,D)**. Increased P-selectin expression was observed in skin collected from the WH + Vehicle group 6 and 12 days after injury **(B,E)**, whereas low levels of this protein were found in mice treated with adelmidrol + *trans*-traumatic acid **(C,F)**. The data are also presented graphically as percentage of total tissue area **(G)**. **(G)** ^∗∗∗^*p* < 0.001 vs. Control; ###*p* < 0.001 vs. WH + Vehicle, as determined through one-way ANOVA followed by the Bonferroni *post hoc* test.

To evaluate whether the treatment with adelmidrol + *trans*-traumatic acid is capable of modulating adhesion molecules, we performed an immunohistochemical staining for ICAM-1 and P-selectin. At 6 and 12 days after wound induction, we observed in the control groups low levels of ICAM-1 (**Figures 10A,D** and relative histological analysis **Figure 10G**) and P-selectin (**Figures 11A,D** and relative histological analysis **Figure 10G**). An increase in ICAM-1 and P-selectin positive stain was found in the WH+Vehicle group both at 6 (**Figures 10B,E** and relative histological analysis **Figure 11G**) and 12 days (**Figures 11B,E** and relative histological analysis **Figure 11G**), whereas the treatment with adelmidrol + *trans*-traumatic acid significantly decreased the levels of ICAM-1 (**Figures 10C,F** and relative histological analysis **Figure 10G**) and P-selectin (**Figures 11C,F** and relative histological analysis **Figure 11G**) both at 6 and 12 days after damage.

### Effect of Adelmidrol + *Trans*-Traumatic Acid on Matrix Metalloproteinase Expression After Wound Induction

**FIGURE 12:**
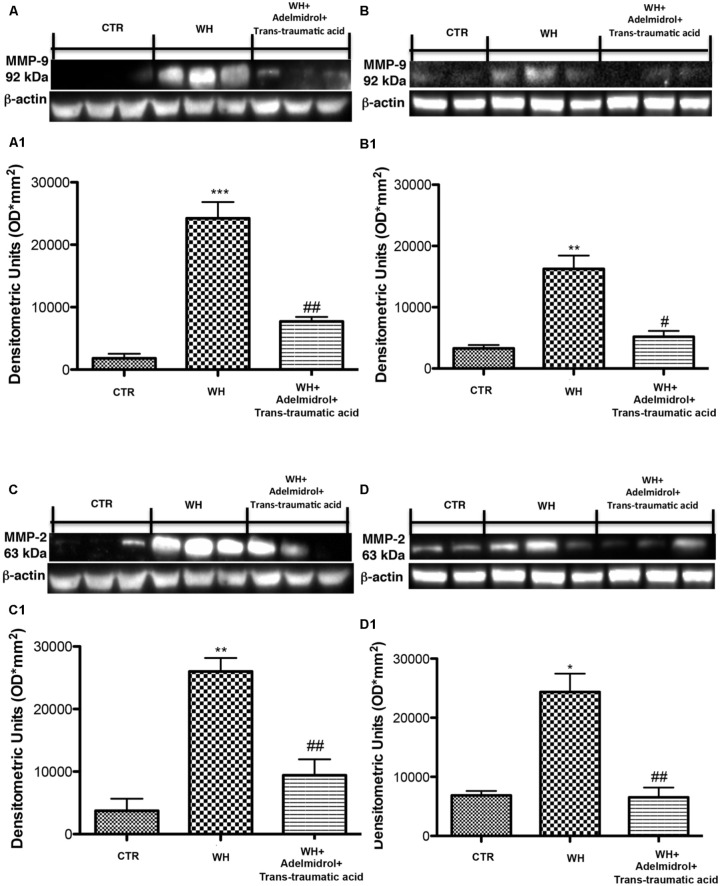
Effects of adelmidrol+trans-traumatic acid on MMP-9 and MMP-2 expression in diabetic mice after 6 and 12 days from wound. Representative western blots showing the effects of adelmidrol + *trans*-traumatic acid on: **(A,A1)** MMP-9 expression, **(C,C1)** MMP-2 expression at 6 days after wounding. Adelmidrol + *trans*-traumatic acid treatment decreased MMP-9 and MMP-2 levels. Data are illustrative of at least three independent experiments. **(A1)** ^∗∗∗^*p* < 0.001 vs. Control; ##*p* < 0.01 vs. WH + Vehicle; **(C1)** ^∗∗^*p* < 0.01 vs. Control; ##*p* < 0.01 vs. WH + Vehicle. Western blot analysis showed the effects of adelmidrol + *trans*-traumatic acid on: **(B,B1)** MMP-9 expression, **(D,D1)** MMP-2 expression at 12 days after wound induction. Adelmidrol + *trans*-traumatic acid treatment reduced MMP-9 and MMP-2 expression. Data are illustrative of at least three independent experiments. Shown is a demonstrative blot of lysates from 10 animals/group, together with a densitometric analysis for all animals. **(B1)** ^∗∗^*p* < 0.01 vs. Control; #*p* < 0.05 vs. WH + Vehicle; **(D1)** ^∗^*p* < 0.05 vs. Control; ##*p* < 0.01 vs. WH + Vehicle. One-way ANOVA followed by the Bonferroni *post hoc* test analyzed data. b-Actin loading control is reused in this figure.

Matrix metalloproteinase are particularly involved in the WH process. By western blot analysis, we demonstrated that MMP-9 and MMP-2 expression was significantly increased after 6 and 12 days from wound induction (**Figures 12A,B** for MMP-9 and see relative densitometric analysis in **Figures 12A1,B1**; **Figures 12C,D** for MMP-2 and see relative densitometric analysis in **Figures 12C1,D1**). A significant reduction of both MMPs was observed after the topical treatment of the wound with adelmidrol + *trans*-traumatic acid for 6 days (**Figures 12A,C** and see relative densitometric analysis in **Figures 12A1,C1**). Moreover, MMP-2 and MMP-9 expression appears to be significantly reduced even after 12 days of treatment with adelmidrol + *trans*-traumatic acid (**Figures 12B,D** and see relative densitometric analysis in **Figures 12B1,D1**).

## Discussion

The skin is the greatest organ in the human body that performs a number of fundamental functions such as temperature regulation and also acts as a barrier to harmful pathogens. The integrity of the skin and its appropriate restoration within the WH process is essential to support these functions. WH involves a complex and closely regulated series of molecular events simplified by an array of cell types, recruited to the place of injury, including keratinocytes, fibroblasts, neutrophils, and macrophages (Martin and Leibovich, 2005; Koh and DiPietro, 2011; Darby et al., 2014).

The process of WH is normally divided into three stages: inflammatory phase, and proliferative and tissue remodeling with scar formation (Clark, 1989). Furthermore, during these stages there are contraction, granulation, epithelialization, and collagenation (Wild et al., 2010).

Compromised WH is a major concern for hospitalization, in particular for diabetic patients in whom their wounds do not heal appropriately and remain at the site exposed to damage.

Adelmidrol is a derivative of azelaic acid and belongs to the ALIAmide family (Aloe et al., 1993). This compound has anti-inflammatory and antinociceptive proprieties similar to PEA (Costa et al., 2008; Genovese et al., 2008). Beneficial effects of adelmidrol have been revealed in numerous preclinical studies (De Filippis et al., 2009; Cordaro et al., 2016; Di Paola et al., 2016; Impellizzeri et al., 2016). Numerous studies displayed that topical application of adelmidrol increased MC granular density, suggesting a diminution in their degranulation (Noli and Miolo, 2001; Cerrato et al., 2012). In addition, this compound exhibited some positive effects in a pilot study on mild atopic dermatitis (Pulvirenti et al., 2007).

At last, the intention of this study was to value the capacity of Nevamast^®^, a gel consisting of 2% adelmidrol and 1% trans-traumatic acid, to improve wound repair in healing-impaired diabetic mouse models. To evaluate the impairment of WH, delay in granulation tissue formation, and reduction of collagen content, we performed a histological analysis and Masson’s trichrome staining. Our results showed that adelmidrol + *trans*-traumatic acid was able to accelerate the healing process in diabetic mice as early as 6 days after the injury and that the re-epithelialization process was almost complete after 12 days, compared with the animals in the WH + Vehicle group that were not treated. Furthermore, the synthesis of collagen was observed already on the sixth day, and its deposition was even higher 12 days after the injury.

Inflammation in the wound is generally caused by resident MCs and precursors of MCs recruited from the circulation, along with monocytes, neutrophils, and T cells that enter the tissue from the blood after damage (Eming et al., 2007; Koh and DiPietro, 2011). In this study, MC infiltration was evaluated using toluidine blue staining. Our results showed that the number of mast cells significantly increased after wound procedure and that this number remained high after 6 and 12 days from the injury. The wounds of diabetic mice treated with adelmidrol + *trans*-traumatic acid showed an important diminution in the number of MCs already on the sixth day.

TGF-β is a family of growth factors intricate in a number of fundamental cellular functions. All three isoforms (TGF-β1, TGF-β2, and TGF-β3) seem to be present in WH. In particular, TGF-β is involved in all stages of WH: inflammation, angiogenesis, fibroblast proliferation, collagen synthesis, and deposition as well as restoration of the new extracellular matrix (Roberts et al., 1986; Nall et al., 1996). After tissue damage, blood vessels breach and the subsequent exposure of platelets to subendothelial collagen produces platelet aggregation, degranulation, and activation of the coagulation cascade. After the hemostasis, TGF next participates as an inflammatory mediator and a strong chemoattractant for several categories of immune cells, including polymorphonuclear and other neutrophil cells (Daniels et al., 2003; Colwell et al., 2005; Sisco et al., 2008) and circulating monocytes (Scott et al., 1995; Sayani et al., 2000; Vial et al., 2011).

Next to their recruitment, it is also notorious that numerous subsequent roles of macrophages, comprising the initiation of granulation tissue formation and angiogenesis, are mediated by TGF (Brandes et al., 1991; Gurtner et al., 2008). Our results demonstrate that, after injury, TGF-β levels were low in diabetic mice both 6 and 12 days after wound induction, whereas treatment with adelmidrol + *trans*-traumatic acid led in both cases to a significant increase in TGF-β expression, highlighting its important role in the WH process in diabetic mice.

Moreover, although still not entirely understood, a role for TGF as a modulator of angiogenesis has been acknowledged (Carmeliet and Jain, 2011). TGF’s capacity to stimulate angiogenesis might be associated with its ability to stimulate VEGF expression at the site of the wound. VEGF intervenes angiogenic activity during the proliferative stage of WH (Nissen et al., 1998), and TGF is notorious to recruit VEGF-generating hematopoietic effector cells to stimulate angiogenesis *in vivo* (Fang et al., 2012).

One of the mechanisms at the base of the impairment of healing in diabetic animals is through the result of a deficiency in VEGF (Altavilla et al., 2001).

Vascular endothelial growth factor has a central function in the induction of angiogenesis based on its capability to stimulate the expression of proteases that digest the components of the extracellular matrix that block angiogenesis, to stimulate endothelial cell proliferation, and to forestall their apoptosis (Griffioen and Molema, 2000).

From our study it was shown how, at 6 days from the damage, adelmidrol + *trans*-traumatic acid stimulated the proliferation of endothelial cells, the secretion of angiogenic cytokines and growth factors, such as VEGF to cause the germination of new blood vessels in the wound bed. We also confirmed this effect by measuring the levels of eNOS expression in the wounds of diabetic mice treated with adelmidrol + *trans*-traumatic acid. However, 12 days after the induction of the wound, a reduction in VEGF and eNOS levels was observed in mice treated with adelmidrol + *trans*-traumatic acid. This difference highlights how adelmidrol + *trans*-traumatic acid is able to accelerate the WH process, because in the mice treated after 12 days the wound is almost completely healed and therefore the intervention of VEGF and eNOS is no longer required.

Endothelial adhesion molecules are crucial in regulatory the migration of circulating leukocytes into peripheral tissue after their damage. Alteration or elimination of these adhesion molecules retards WH by decreasing leukocyte infiltration and attenuating the inflammatory reaction (Nagaoka et al., 2000).

Leukocyte recruitment into inflammatory places is obtained by using different inducible or constitutive adhesion molecules (Butcher, 1991; Ley and Tedder, 1995; Springer, 1995). L-selectin is constitutively expressed by a majority of leukocytes, whereas activated endothelial cells express E-selectin and P-selectin that primarily mediate leukocyte rolling on the endothelium (Tedder et al., 1995). ICAM-1 is constitutively expressed by endothelial cells, and it is quickly upregulated during inflammation, resulting in increased leukocyte-endothelial cell adhesion (Dustin et al., 1986).

From our study, an alteration of the expression of these two proteins has been observed, which affects the delay of WH in diabetic mice. Treatment with adelmidrol + *trans*-traumatic acid was able to reduce this alteration and therefore to accelerate the healing of wounds both after 6 and 12 days from the induction of the wound. MMPs are a family of zinc-dependent endopeptidases that degrade approximately all extracellular matrix and basement membrane proteins at a neutral pH. The MMPs play an important role in numerous pathologic and biologic processes, with their involvement in WH being first revealed in guinea pigs (Grillo and Gross, 1967). Their most important function is degradation, by elimination of damaged extracellular matrix during the inflammatory stage, rupture of the capillary basement membrane for angiogenesis and cell migration during the proliferation stage, and contraction and remodeling of tissue in the remodeling stage. The disequilibrium in MMPs might increase the chronicity of a lesion, a common problem found in diabetic patients. Death et al. (2003) discovered that hyperglycemia disturbed the regulation of MMP/TIMP and increased the activities of MMP-9, MMP-2 and MMP-1 in vascular cells, stimulating the degradation of the extracellular matrix and producing an imbalance in diabetes. Our results have clarified that an increase of MMP-2 and MMP-9 expression may be a factor resulting in impairment of WH, while topical treatment with adelmidrol + *trans*-traumatic acid prevented overexpression of both MMPs by favoring WH in diabetic mice 6 and 12 days after the injury.

## Conclusion

We demonstrated that adelmidrol + *trans*-traumatic acid treatment accelerates skin WH in diabetic mice by inducing the expression of numerous factors involved in different stages of the healing process. Skin WH in diabetic patients is a particularly challenging clinical problem. Adelmidrol + *trans*-traumatic acid treatment can be considered a promising therapeutic option in these patients.

## Data Availability

The authors declare that all data supporting the findings of this study are available within the article. The data that support the findings of this study are available from the corresponding author upon reasonable request.

## Ethics Statement

The University of Messina Review Board for the care of animals approved the research. All animal experiments observe the regulations in Italy (D. M. 116192) as well as the EU regulations (O. J. of E. C. L 358/1 12/18/1986).

## Author Contributions

RS, DI, and SC conceptualized and designed the study. RS, DI, MC, EG, and AP acquired the data. RS, DI, MC, RDP, and SC analyzed and interpreted the data. RS and DI drafted the manuscript. RS, DI, MC, and SC performed the statistical analysis. SC and RDP critically revised the manuscript for intellectual content. All the authors read and approved the final manuscript.

## Conflict of Interest Statement

SC is a co-inventor on patent WO2013/121449 A8 (Epitech Group SpA); methods for the modulation of amidases capable of hydrolyzing *N*-acylethanolamines useable in the therapy of inflammatory diseases. Moreover, SC is also a co-inventor with Epitech group on the following patents: EP 2814489; EP 2821083; EP 2985037; 102015000067344. The remaining authors declare that the research was conducted in the absence of any commercial or financial relationships that could be construed as a potential conflict of interest.
